# Risk of mortality associated with concomitant antidepressant and benzodiazepine therapy among patients with depression: a population-based cohort study

**DOI:** 10.1186/s12916-020-01854-w

**Published:** 2020-12-09

**Authors:** Han Eol Jeong, Ha-Lim Jeon, In-Sun Oh, Woo Jung Kim, Ju-Young Shin

**Affiliations:** 1 School of Pharmacy, Sungkyunkwan University, Suwon, Gyeonggi-do South Korea; 2 Department of Psychiatry, Yongin Severance Hospital, Yonsei University College of Medicine, Yongin, Gyeonggi-do South Korea; 3 Institute of Behavioral Sciences in Medicine, Yonsei University College of Medicine, Seoul, South Korea; 4 Samsung Advanced Institute for Health Sciences & Technology, Sungkyunkwan University, Seoul, South Korea

**Keywords:** Antidepressants, Benzodiazepines, Concomitant therapy, Depression

## Abstract

**Background:**

With antidepressants (ADs) having minimal therapeutic effects during the initial weeks of treatment, benzodiazepines (BZDs) are concomitantly used to alleviate depressive symptoms of insomnia or anxiety. However, with mortality risks associated with this concomitant use yet to be examined, it remains unclear as to whether this concomitant therapy offers any benefits in treating depression.

**Methods:**

We conducted a population-based cohort study using South Korea’s nationwide healthcare database from 2002 to 2017. Of 2.6 million patients with depression, we identified 612,729 patients with incident depression and newly prescribed ADs or BZDs, by excluding those with a record of diagnosis or prescription within the 2 years prior to their incident diagnosis. We classified our study cohort into two discrete groups depending on the type of AD treatment received within 6 months of incident diagnosis—AD monotherapy and AD plus BZD (AD+BZD) therapy. We matched our study cohort in a 1:1 ratio using propensity scores to balance baseline characteristics and obtain comparability among groups. The primary outcome was all-cause mortality, and patients were followed until the earliest of outcome occurrence or end of the study period. We conducted multivariable Cox proportional hazards regression analysis to estimate adjusted hazards ratios (HRs) with 95% confidence intervals (CIs) for the risk of mortality associated with AD+BZD therapy versus AD monotherapy.

**Results:**

The propensity score-matched cohort had 519,780 patients with 259,890 patients in each group, where all baseline characteristics were well-balanced between the two groups. Compared to AD monotherapy, AD+BZD therapy was associated with an increased risk of all-cause mortality (adjusted HR, 1.04; 95% CI, 1.02 to 1.06).

**Conclusions:**

Concomitantly initiating BZDs with ADs was associated with a moderately increased risk of mortality. Clinicians should therefore exercise caution when deciding to co-prescribe BZDs with ADs in treating depression, as associated risks were observed.

**Supplementary information:**

**Supplementary information** accompanies this paper at 10.1186/s12916-020-01854-w.

## Background

Depression is a common psychiatric illness that affects > 300 million patients worldwide [1]. Accordingly, the utilization of antidepressants (ADs) has increased over time as well [2–5]. With ADs having minimal therapeutic effects during the initial weeks of administration [6], benzodiazepines (BZDs) are often additionally administered to manage anxiety or insomnia in patients with depression [7, 8]; one in 10 patients who initiated ADs concomitantly initiated BZDs in the USA [9]. However, BZDs are sometimes continued for longer periods than intended in real-world clinical practice, possibly owing to their dependency—one study found that approximately 12% of patients who received concomitant BZD and AD therapy (AD+BZD) continued long-term BZD use [9]. Despite such prevalence, uncertainties remain regarding the safety of AD+BZD therapy for the treatment of patients with depression.

To our knowledge, no previous study, observational or randomized controlled trial, has assessed the risk of mortality associated with AD+BZD therapy, as compared with AD therapy alone, among depressed patients. With no consensus on the potential benefits or harms of BZDs when used with ADs, clinical guidelines [10, 11] have expressed concerns regarding prolonged BZD use, especially as several studies reported an increased risk of mortality associated with it [12, 13]. Moreover, a meta-analysis of randomized trials found that adults with depression who received AD+BZD therapy reported more adverse events than those receiving ADs alone [7]. However, as randomized trials are generally more focused on assessing the medication’s efficacy, observational studies are needed to make a formal assessment regarding mortality. Meanwhile, one meta-analysis found that AD+BZD therapy could potentially improve depression severity and remission compared to AD therapy alone [14]. To date, the benefit or harm associated with AD+BZD therapy remains uncertain with limited real-world evidence available on its use.

Therefore, this nationwide cohort study aimed to investigate the risk of mortality associated with concomitant AD+BZD therapy when compared to AD monotherapy among patients with incident depression.

## Methods

### Data source

We used the National Health Insurance Service-National Health Insurance Database (NHIS-NHID) of South Korea [15], which contains health insurance claims data for the entire Korean population of 50 million inhabitants collected between 1 January 2002 and 31 December 2017. The NHIS-NHID is comprised of anonymized patient identifier and corresponding information on associated sociodemographic characteristics (age, sex, health insurance type, and income level), healthcare utilization history, diagnostic codes based on the International Classification of Diseases 10th Revision (ICD-10), and drug prescription information (national drug codes [NDC], days’ supply, dosage, and administration route). The NDCs are based on the drug’s active ingredient and mapped to the World Health Organization’s Anatomical Therapeutic Chemical classification codes. Moreover, the date of death was linked to the national vital statistics maintained by Statistics Korea.

### Study population

**Fig. 1:**
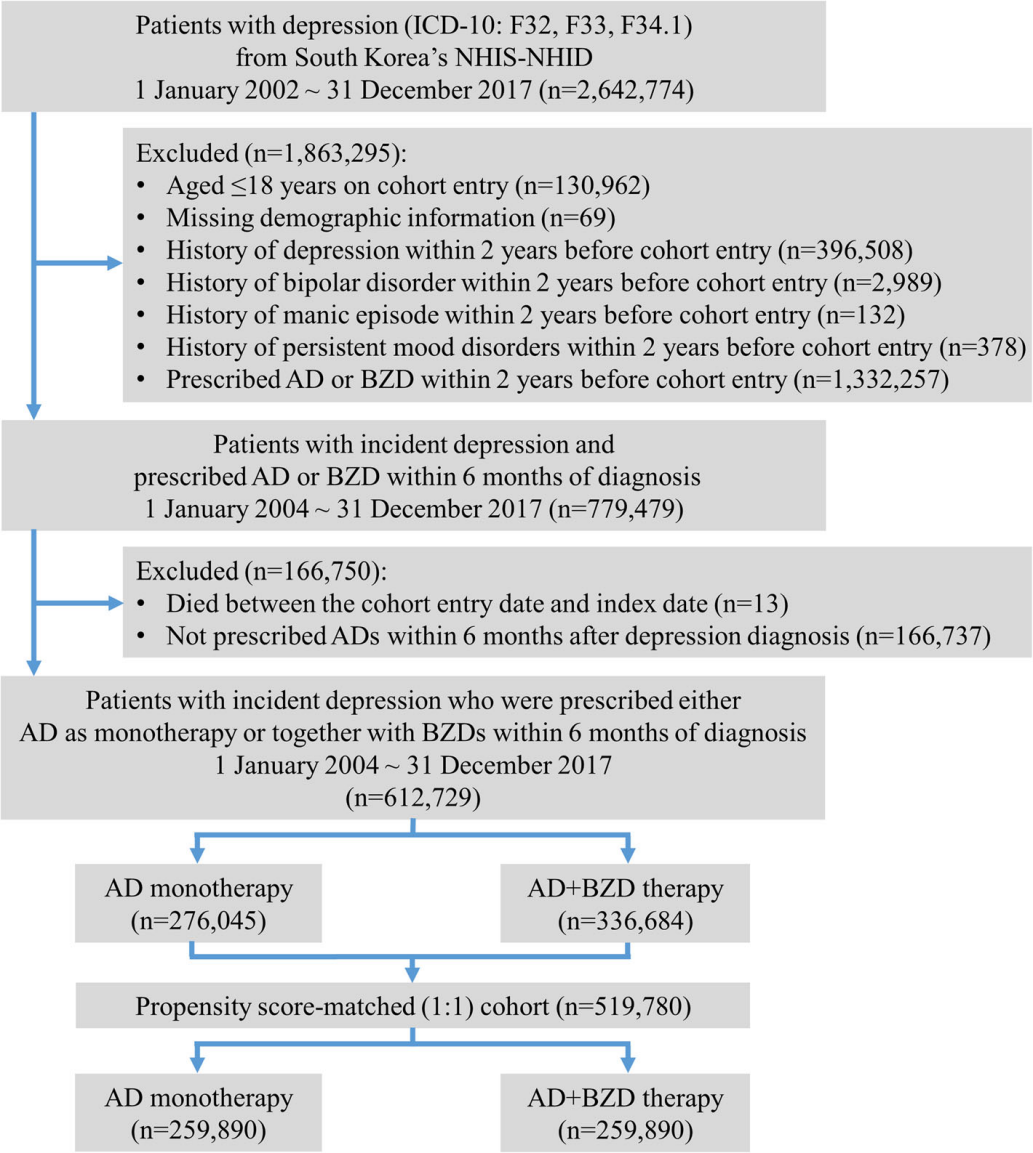
Study cohort flowchart. *Study outcomes comprised all-cause mortality, suicide attempt/self-harm, or all-cause hospitalization. Note: AD, antidepressants; BZD, benzodiazepines; NHIS-NHID, National Health Insurance Service-National Health Insurance Database

We identified patients with either a primary or secondary recorded diagnosis of incident depression (ICD-10: F32, F33, F34.1) from both inpatient and outpatient settings between 1 January 2004 and 31 December 2017 (Fig. 1). Cohort entry was defined as the date of incident diagnosis with depression. The following patients were excluded from our study: (1) those diagnosed with depression (ICD-10: F32, F33), bipolar disorders (F31), manic episodes (F30), or persistent mood disorders (F34) within 2 years prior to cohort entry, to restrict the analysis to incident patients with depression; (2) those prescribed any ADs or BZDs within 2 years prior to cohort entry to restrict to new users of ADs or BZDs; (3) those aged < 18 years at cohort entry as depression is unlikely to be prevalent and because AD or BZD use is not advised in this age group; and (4) missing demographic information.

### Exposure definition

We used prescription records of ADs and BZDs from both inpatient and outpatient settings to ascertain exposure, where an intention-to-treat approach was used to define follow-up (Additional file 1: Table S1). Prescriptions of ADs or BZDs within 6 months after cohort entry were eligible. Exposure was classified into two groups and their corresponding index dates were defined as follows: (1) AD monotherapy, date when one class of AD was prescribed in a single prescription; (2) AD+BZD therapy, date when ADs were prescribed together with BZDs in a single prescription on the same day (Additional file 2: Fig. S1 and Additional file 3: Fig. S2).

### Outcome definition

Time to all-cause mortality was our primary outcome of interest. Our secondary outcomes were the time to incident diagnosis with suicide attempt/self-harm (from both inpatient and outpatient settings) and time to all-cause hospitalization, defined as a visit to a medical institution resulting in admission. Follow-up began on the index date and ended on the earliest of the outcome occurrence, or end of the study period (31 December 2017).

### Potential confounders

Age, sex, health insurance type, residential district, and income level were assessed at cohort entry. Furthermore, comorbidities (anxiety, cancer, cerebrovascular disease, chronic kidney disease, chronic obstructive pulmonary disease, dementia, diabetes mellitus, epilepsy, fractures, hypertension, hyperlipidemia, insomnia, ischemic heart disease, osteoarthritis, Parkinson’s disease, rheumatoid arthritis, and substance abuse) and history of medication use (angiotensin-converting-enzyme inhibitors, angiotensin II receptor blockers, anticholinergics, antiplatelets and anticoagulants, antipsychotics, anticonvulsants, digoxin, non-insulin glucose-lowering agents, anti-inflammatory analgesics, beta-blockers, calcium channel blockers, insulin, lipid-lowering agents, narcotic analgesics, nonsteroidal anti-inflammatory drugs, other anxiolytics, and thiazide diuretics) were assessed within the year before cohort entry. Comorbidities were defined using ICD-10 codes and use of medications was defined using NDCs (Additional file 1: Table S1). The Charlson comorbidity index (CCI) score was also estimated to determine the overall comorbidity burden [16].

To obtain comparability between treatment groups, we conducted propensity score (PS) matching, where the PS of receiving AD therapy was estimated using multivariable logistic regression. Upon conducting chi-square tests to determine statistical significance between each confounder and all-cause mortality, only confounders that had *p* value < 0.2 were included as independent variables into the multivariable logistic regression model (history of other anxiolytic use was not included in the model) [17]; age and sex were always included regardless to their *p* value. Matching based on PS was done in a 1:1 ratio for the two groups using the Greedy 5→1 digit matching macro [18, 19], where the *c* statistic (0.6–0.8) was used to assess model discrimination [20].

### Statistical analyses

The baseline characteristics of our study subjects are represented as counts (proportions) and means (standard deviations) for categorical and continuous variables, respectively. We estimated the absolute standardized difference (aSD) to determine imbalances between groups (aSD > 0.1 indicated an important imbalance) [21].

We first aimed to examine the risk of study outcomes by calculating the incidence of study outcomes per 1000 person-years and estimating the absolute risk and risk differences with 95% confidence intervals (CIs). Adjusted hazard ratios (HRs) with 95% CIs for study outcomes associated with AD+BZD therapy versus AD monotherapy were estimated using multivariable Cox proportional hazards regression, adjusted for all potential confounders. We tested for the proportional hazards assumption by using the Schoenfeld residuals and found a *p* value of 0.0003. As the proportional hazards assumption was violated and, thus, indicated non-proportional hazards, we stratified into different, non-overlapping time periods in 1-year intervals to utilize all available follow-up. Moreover, we plotted the cumulative incidence for the risk of study outcomes associated with the type of AD therapy received.

### Subgroup analyses

Stratification was based on age (< 65 years versus ≥ 65 years), and sex, where a single model with interaction terms, was used to observe whether the association between exposure and outcome differed significantly among subgroups. Moreover, among AD+BZD recipients, we stratified on the type of BZD received (short-acting versus long-acting; patients who received both BZD types with antidepressants were excluded in this subgroup analysis) and patients who discontinued BZDs, defined as those who did not use benzodiazepines continuously for 6 months (180 days) after the index date; this definition was previously used by several studies and is considered a common definition [9, 22]. Benzodiazepine treatment length was defined using a 30-day grace period that was added to the days’ supply to allow for gaps in between prescriptions.

### Sensitivity analyses

First, we varied the definition of concomitant therapy to at least one prescription of BZD within 2 weeks of the first AD prescription (Additional file 4: Fig. S3). Second, exposure was ascertained using an as-treated approach, where patients were censored when they have either discontinued treatment (defined as when no new AD or BZD prescription was given within 30 days of the end of the previous prescription [23]) or added BZDs throughout follow-up among AD monotherapy recipients. Third, we applied two other PS methods of inverse probability of treatment weighting and model adjustment [19, 24]. Lastly, we estimated the *E*-value to assess the potential impact of unmeasured confounders on our study findings [25], where in brief, a large *E*-value would imply that large unmeasured confounding would be needed to explain the observed association. All analyses were performed using the SAS Enterprise Guide program provided by the NHIS (Release 9.71, SAS Institute Inc., Cary, NC, USA).

## Results

Of 2,642,774 patients with depression, we identified 612,729 patients to be included in our study cohort. Among the 519,780 patients in our 1:1 PS-matched cohort, 259,890 received AD monotherapy and AD+BZD therapy, respectively (*c* statistics: 0.585 for AD monotherapy versus AD+BZD therapy) (Fig. 1). Of AD+BZD therapy recipients prior to PS matching (*n* = 336,684), 81.5%, 17.5%, and 1.0% received either 1, 2, and ≥ 3 antidepressant(s) with a benzodiazepine. Baseline characteristics were well balanced after matching, as all variables had an aSD < 0.1 (Table 1 and Additional file 5: Table S2).

**Table 1:** Baseline characteristics of study subjects after propensity score matching, where values are percentages unless stated otherwise

	Propensity score-matched cohort (1:1 match)				
	***N*** = 519,780 (%)				
	AD monotherapy		AD + BZD therapy		aSD
	***N*** = 259,890 (%)		***N*** = 259,890 (%)		
**Follow-up (years; mean ± std)**	5.56	± 3.86	6.24	± 4.15	0.169
**Age (years; mean ± std)**	43.9	± 17.1	43.4	± 16.1	0.027
**Male**	112,411	43.25	113,154	43.54	0.006
**Type of insurance**					0.000
Healthcare insurance	250,074	96.22	250,233	96.28	
Medical aid	9595	3.69	9449	3.64	
**Residential district**					0.000
Metropolitan	139,908	53.83	140,636	54.11	
Urban	44,264	17.03	44,499	17.12	
Rural	75,090	28.89	74,159	28.53	
**Income level**					0.000
1st quartile	46,610	17.93	46,059	17.72	
2nd quartile	51,464	19.80	51,229	19.71	
3rd quartile	62,992	24.24	62,850	24.18	
4th quartile	83,323	32.06	84,424	32.48	
**Charlson Comorbidity Index (mean ± std)**	0.36	± 0.78	0.35	± 0.78	0.011
0	202,809	78.04	204,010	78.50	
1	31,040	11.94	30,658	11.80	
2	17,963	6.91	17,425	6.70	
3	7541	2.90	7272	2.80	
4	149	0.06	133	0.05	
≥ 5	388	0.15	392	0.15	
**Comorbidities**^**†**^					
Anxiety	4618	1.78	4560	1.75	0.002
Cancer	3545	1.36	3468	1.33	0.003
Cerebrovascular disease	4558	1.75	4482	1.72	0.002
Chronic kidney disease	619	0.24	607	0.23	0.001
Chronic obstructive pulmonary disease	8930	3.44	8659	3.33	0.006
Dementia	1997	0.77	2012	0.77	0.001
Diabetes mellitus	15,116	5.82	14,741	5.67	0.006
Epilepsy	1071	0.41	1037	0.40	0.002
Fractures	1327	0.51	1299	0.50	0.002
Hypertension	27,875	10.73	26,808	10.32	0.013
Hyperlipidemia	26,392	10.16	25,583	9.84	0.010
Insomnia	7784	3.00	7547	2.90	0.005
Ischemic heart disease	1534	0.59	1496	0.58	0.002
Osteoarthritis	21,739	8.36	21,200	8.16	0.008
Parkinson’s disease	221	0.09	201	0.08	0.003
Rheumatoid arthritis	3656	1.41	3565	1.37	0.003
Substance abuse	944	0.36	1021	0.39	0.005
**History of medication use**^**†**^					
Angiotensin-converting-enzyme inhibitors	2036	0.78	1961	0.75	0.003
Angiotensin II receptor blockers	15,077	5.80	14,664	5.64	0.007
Anticholinergics	25,843	9.94	25,751	9.91	0.001
Antiplatelets and anticoagulants	19,241	7.40	18,730	7.21	0.008
Antipsychotics	1997	0.77	1926	0.74	0.003
Anticonvulsants	4345	1.67	4363	1.68	0.001
Digoxin	340	0.13	345	0.13	0.001
Non-insulin glucose-lowering agents	8765	3.37	8607	3.31	0.003
Anti-inflammatory analgesics	101,017	38.87	100,937	38.84	0.001
β-blockers	10,291	3.96	9616	3.70	0.014
Calcium channel blockers	16,733	6.44	16,063	6.18	0.011
Insulin	885	0.34	872	0.34	0.001
Lipid-lowering agents	14,689	5.65	14,207	5.47	0.008
Narcotic analgesics	62,273	23.96	61,439	23.64	0.008
Nonsteroidal anti-inflammatory drugs	139,850	53.51	139,982	53.84	0.001
Other anxiolytics	14,899	5.73	14,851	5.71	0.001
Thiazide diuretics	10,403	4.00	9949	3.83	0.009

*AD* antidepressant, *aSD* absolute standardized difference, *BZD* benzodiazepine, *std* standard deviation

^**†**^Assessed within the year before cohort entry

**Fig. 2:**
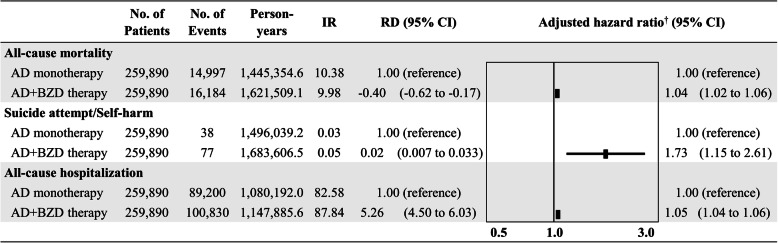
Risk of all-cause mortality, suicide attempt/self-harm, and all-cause hospitalization for antidepressant monotherapy and antidepressant+benzodiazepine concomitant therapy in the propensity score-matched cohort. Note: AD, antidepressant; BZD, benzodiazepine; CI, confidence interval; IR, incidence rate; RD, risk difference. *Incidence rate = (number of events/total person-years) × 1000. ^†^Adjusted for age, sex, CCI, comorbidities, and concomitant medications

**Fig. 3:**
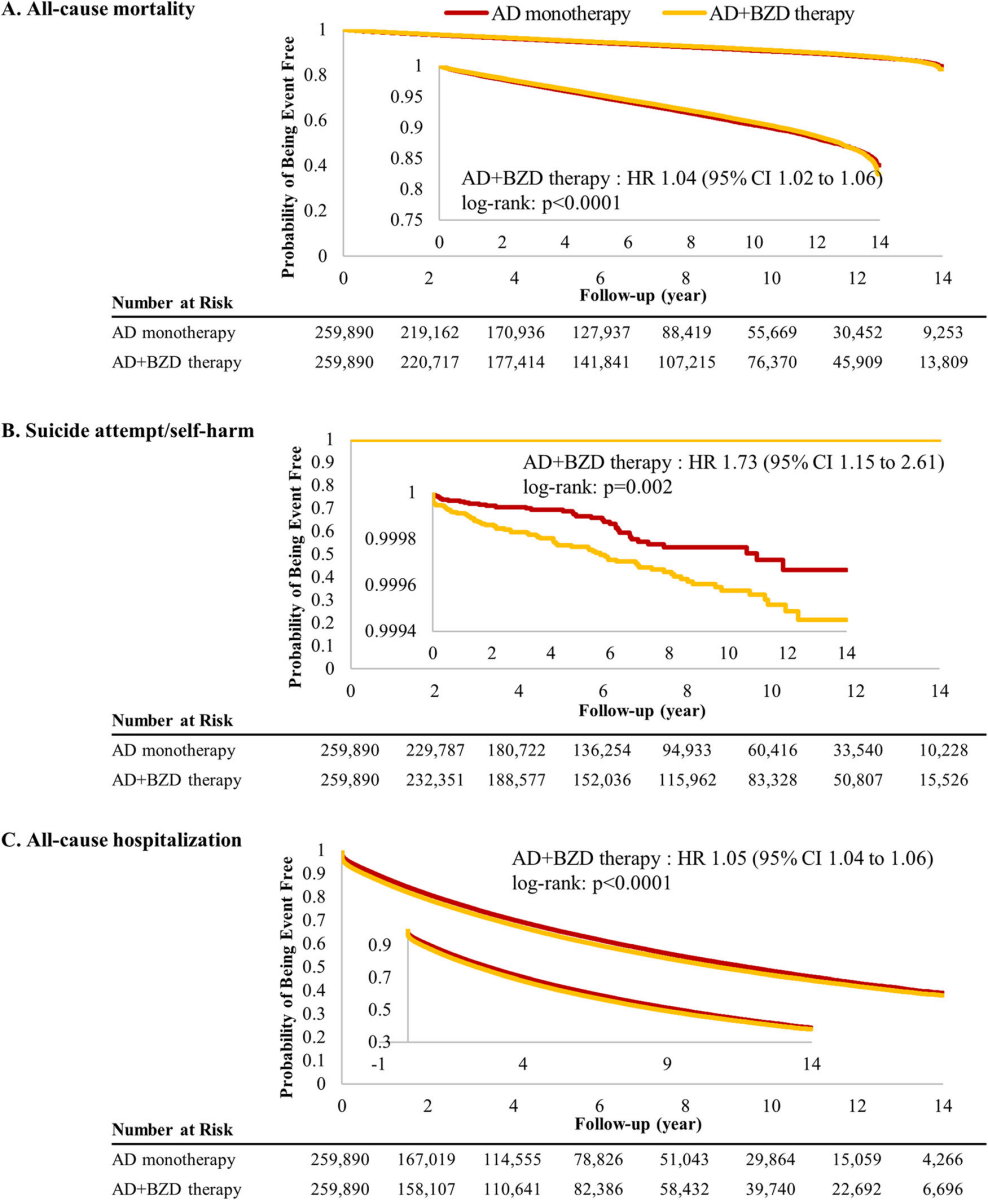
Kaplan-Meier survival curve of all-cause mortality, suicide attempt/self-harm, and all-cause hospitalization

AD+BZD therapy was associated with a moderately increased risk of all-cause mortality (adjusted HR, 1.04; 95% CI, 1.02 to 1.06) and all-cause hospitalization (adjusted HR, 1.05; 95% CI, 1.04 to 1.06) when compared to AD monotherapy; the top 20 frequent causes of hospitalization are shown in Additional file 6: Table S3. In contrast, the risk of suicide attempt/self-harm was significant increased with AD+BZD therapy (adjusted HR, 1.73; 95% CI, 1.15 to 2.61) as compared with AD monotherapy (Figs. 2 and 3).

**Fig. 4:**
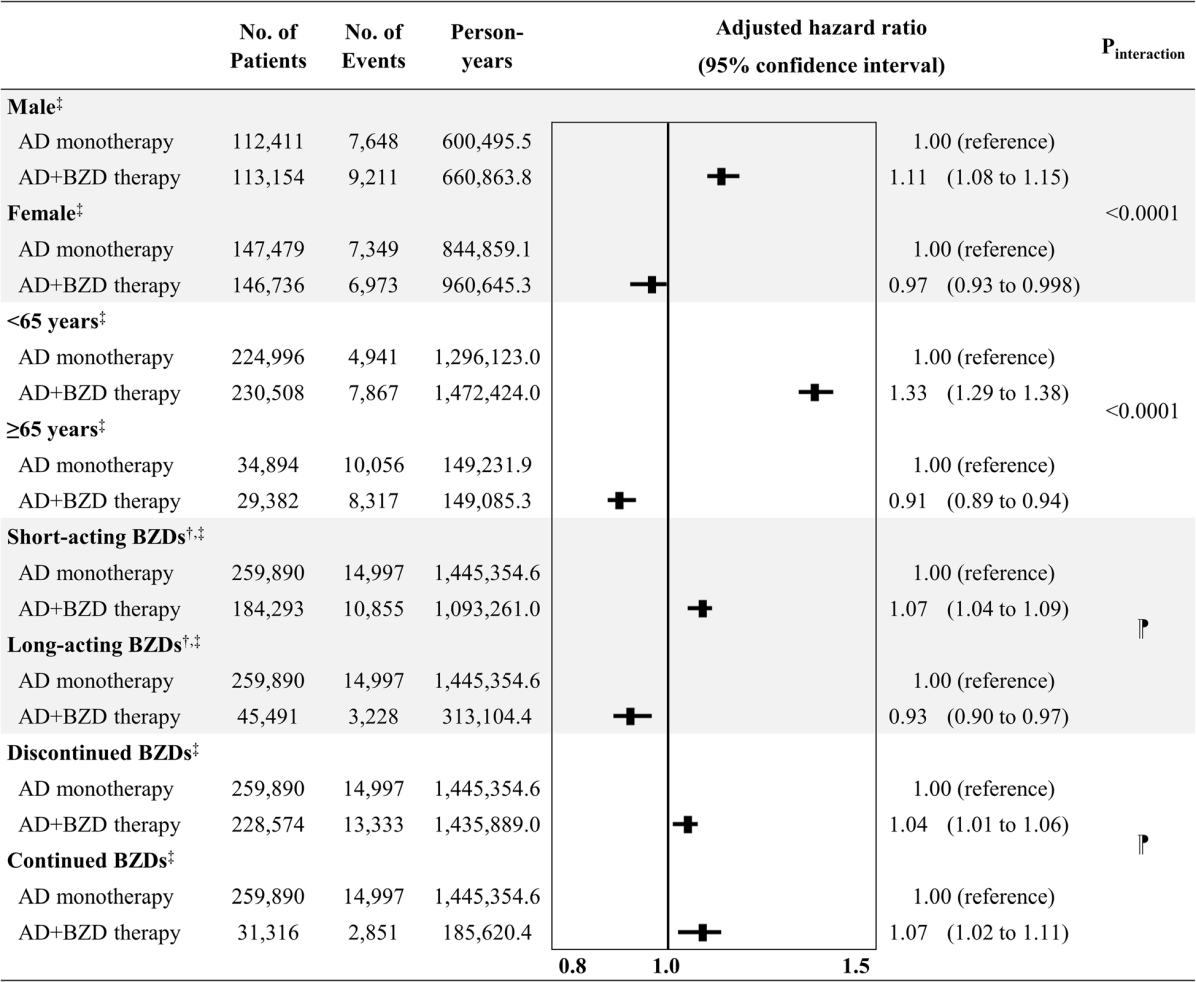
Forest plot summarizing the risk of all-cause mortality for antidepressant monotherapy and antidepressant+benzodiazepine concomitant therapy after stratifying for age, sex, type of benzodiazepines, and benzodiazepine discontinuation. Note: AD, antidepressants; aSD, absolute standardized difference; BZD, benzodiazepines. ^†^Patients who received both short- and long-acting BZDs together with antidepressants on the index date were not considered for in this subgroup analysis as they were unable for classification. ^‡^Sex-stratified analysis: all covariates remained balanced among females as all aSD values were < 0.1, whereas males showed imbalance in health insurance type (aSD 0.152) and residential district (aSD 0.143); age-stratified analysis: all covariates remained balanced among those < 65 years, while CCI (aSD 0.164) and history of NSAIDs use (aSD 0.115) were imbalanced among those ≥ 65 years; stratified for BZD type: all covariates remained balanced among those who received short-acting BZDs but showed imbalance in residential district (aSD 0.180) and CCI (aSD 0.147) among those who received long-acting BZDs; stratified for discontinuation of BZD: all covariates remained balanced among those who discontinued BZDs, while age (aSD 0.169), health insurance type (aSD 0.132), income level (aSD 0.120), and history of insomnia (aSD 0.107) showed imbalance among those who continued BZDs at 6 months after the index date. ^⁋^P-for-interaction not estimated as subtype of BZDs was our exposure of interest

Significant effect modifications were found when stratified for age and sex (*p* for interaction < 0.0001), as the risk of all-cause mortality was increased among males for AD+BZD therapy (adjusted HR, 1.11; 95% CI, 1.08 to 1.15) but moderately reduced among females (adjusted HR, 0.97; 95% CI, 0.93 to 0.998). Moreover, age-stratified analyses showed that among those aged < 65 years, AD+BZD therapy was associated with an increased risk of all-cause mortality (adjusted HR, 1.33; 95% CI, 1.29 to 1.38), but showed reduced risk in those aged ≥ 65 years (adjusted HR, 0.91; 95% CI, 0.89 to 0.94). Among AD+BZD therapy recipients, those who received short-acting BZDs showed a slightly increased risk of mortality (adjusted HR, 1.07; 95% CI, 1.04 to 1.09), whereas those who received long-acting BZDs showed a 7% reduced risk (adjusted HR, 0.93; 95% CI, 0.90 to 0.97). Finally, similarly increased risk of mortality was observed in those who discontinued BZDs (adjusted HR, 1.04; 95% CI, 1.01 to 1.06) and continued BZDs (adjusted HR, 1.07; 95% CI, 1.02 to 1.11) (Fig. 4). Results of sensitivity analyses remained largely consistent with our main findings (Additional file 7: Fig. S4).

## Discussion

Of 2.6 million patients diagnosed with depression in South Korea between 2002 and 2017, this nationwide cohort study found a moderate 4% increased risk of all-cause mortality with AD+BZD therapy, as compared to AD monotherapy, but a 74% increased risk of suicide attempt/self-harm. Subgroup analyses revealed an elevated risk of all-cause mortality associated with AD+BZD therapy among males, those aged < 65 years, and those who received short-acting BZDs with antidepressants. To our knowledge, this large-scale observational study is the first study conducted using nationwide data to provide real-world evidence that initiating BZDs with AD is associated with potentially fatal harms in the treatment of depression.

No study to date has assessed the risk of mortality associated with AD+BZD therapy versus AD monotherapy among adults with depression. However, one meta-analysis of randomized trials that examined the association between adverse events and AD+BZD therapy was available for an indirect comparison. We assumed these adverse events to have been severe, as their occurrence resulted in patients dropping out of the trial. Comparable to our observed increased risk of mortality associated with AD+BZD therapy, this meta-analysis found that the AD+BZD group was more likely to report ≥ 1 adverse events when compared to the AD monotherapy group (pooled risk ratio, 1.12; 95% CI, 1.01–1.23) [8]. Based on our abovementioned assumption on adverse events, they could serve as surrogate measures or precursors to either mortality or hospitalizations. Moreover, several systematic reviews of observational studies and randomized trials found BZD use to be positively associated with suicides [26, 27], accidents [28], or falls [29]. In addition to these events increasing the risk of death, the risks of suicide attempts and mortality were also higher among patients with depression and anxiety compared to those with depression alone [26, 30]. Thus, recipients of AD+BZD therapy in our study are likely to be depressed patients exhibiting comorbid anxiety. While administering BZDs may alleviate anxiety, it simultaneously puts these patients at higher risks of adverse events (hospitalization or mortality). With no published evidence available, to the best of our knowledge, for direct comparison, our findings in the meantime suggest the potential need in revisiting the rationale behind co-prescribing BZDs with ADs to treat depression, as this clinical practice was associated with a moderately elevated risk of mortality.

Our findings suggest that AD+BZD therapy should be carefully used across all age groups, which are supported by both our main and age-stratified analyses showing an increased risk of all-cause mortality. Although we observed a statistically insignificant risk of mortality associated with AD+BZD therapy among patients ≥ 65 years of age, use of AD+BZD therapy is not warranted in the elderly as BZDs were previously reported to have deleterious effects in this age group [12, 13]. Thus, in addition to their rather well-known harmful effects among the elderly, BZDs should also be used with caution in young and middle-aged adults with incident depression. In the meantime, healthcare providers should consider ways to minimize the use of AD+BZD therapy as the harms appear to outweigh its benefits in treating adults with incident depression across all age groups.

Our study showed that 54.9% (before PS matching) of adults with incident depression received AD+BZD therapy, which was comparable to those (40–50%) in previous studies on other Asian [31, 32] or Caucasian populations [33]. While the clinical manifestation of depression in Korean patients could have been different from that of patients of other countries, cultures, or ethnicities [34], we found similar proportions of AD+BZD use among patients with depression. Moreover, 12% of AD+BZD recipients (*n* = 31,316) in our study continued BZDs at 6 months after follow-up. Despite this observation not agreeing with current guidelines that recommend BZD use to not exceed 4 weeks [35], we believe that the prolonged use of BZDs may have been due to either its own dependence or because concomitant use of BZDs with ADs in treating incident depression may have partially contributed to dependence [36].

This study has several strengths. To the best of our knowledge, this is the first study to have examined the risk of mortality associated with two treatment options commonly used to treat early-stage depression. Second, we used a nationwide South Korean healthcare database, highly representative of the entire South Korean population, to identify 2.6 million adults with depression. As NHIS-NHID provides data to external investigators after rigorous internal review for data validity, it is considered an extremely reliable data source. Third, misclassification of study outcomes is unlikely to have occurred in our study as all records of death in the NHIS-NHID were linked with South Korea’s national vital statistics, which are maintained by Statistics Korea [15]. Lastly, we applied PS matching that included numerous covariates to obtain comparability and balance between the two treatment groups. We also applied two other methods of PS that utilized all patients from our study cohort and found a consistently elevated risk of mortality associated with AD+BZD therapy.

Our study also has limitations. First, there may have been exposure misclassification as recipients of concomitant therapy were classified based on prescriptions made on the same day. However, we expect exposure misclassification to be minimal in our study, as the sensitivity analysis that varied the definition of concomitant therapy showed consistent findings. Second, our main findings, which used the intention-to-treat approach, may have underestimated the risk of study outcomes, as patients would have remained in the study regardless of their treatment patterns throughout follow-up, which may have led to an overestimation of person-time. However, the results of our sensitivity analysis that used the as-treated approach (censored follow-up at treatment discontinuation) were consistent. Third, despite accounting for various confounding factors and using three different PS methods, residual confounding from unmeasured or unaccounted confounders may still be present. However, residual confounding from unmeasured confounders is likely to be small as our HR estimate for mortality was close to the null, which is also supported from the estimated *E*-value that suggests that an unmeasured confounder has to be associated with both the exposure and outcome by greater than 1.24-fold in order to affect the observed association (Additional file 8: Table S4). We therefore suggest that future studies should consider clinical features, for instance, the main symptoms, severity of depression, and suicidal ideation, factors that could not be measured in this study due to structural limitations, possibly by using linked data between health insurance claims and electronic medical records of hospitals and clinics. This would then be able to provide better understanding on the observed protective effects of AD+BZD therapy on mortality risks among those aged ≥ 65 years, females, or those who received long-acting BZDs.

## Conclusions

In conclusion, the addition of BZD to AD monotherapy to treat depression was associated with a moderately increased risk of all-cause mortality when compared to AD monotherapy. This may be due to the risks associated with BZDs; therefore, careful consideration is warranted when deciding to concomitantly initiate BZDs with ADs. While further studies accompanied with more detailed clinical data could provide insight into the underlying effect modifications by gender or age, our findings suggest healthcare providers to exercise caution in co-prescribing BZDs and ADs to treat patients with depression, weighing the risk-benefits associated with concomitant AD+BZD therapy over AD monotherapy.

## Supplementary Information

**Additional file 1: Table S1.** Drug and diagnosis codes used in the study.

**Additional file 2: Fig. S1.** Overall study design.

**Additional file 3: Fig. S2.** Exposure classification.

**Additional file 4: Fig. S3.** Varying the definition of the time-window for concomitant therapy.

**Additional file 5: Table S2.** Baseline characteristics of study subjects before propensity score matching, where values are percentages unless stated otherwise.

**Additional file 6: Table S3.** Top 20 frequent causes of hospitalization by ICD-10 diagnosis code to 3 places.

**Additional file 7: Fig. S4.** Forest plot summarizing the results of sensitivity analyses for the risk of all-cause mortality.

**Additional file 8: Table S4.** Results of sensitivity analyses that examined the effects from unmeasured confounders on the association between risk of mortality and the use of antidepressants with benzodiazepines versus antidepressants alone.

## Data Availability

The data that support the findings of this study are available from the National Health Insurance Service of South Korea but restrictions apply to the availability of these data due to domestic laws and regulations that prohibit the distribution or release of individual’s data to the public, and so are not publicly available. Data are however available from the authors upon reasonable request and with permission of the National Health Insurance Service of South Korea.

## Abbreviations

AD: Antidepressants
aSD: Absolute standardized difference
BZD: Benzodiazepines
CCI: Charlson Comorbidity Index
CI: Confidence interval
HR: Hazard ratio
NHIS-NHID: National Health Insurance Service-National Health Insurance Database
