# Supplementary material for: Risk of mortality associated with concomitant antidepressant and benzodiazepine therapy among patients with depression: a population-based cohort study
Source: BMC Med. 2020 Dec 9;18:387. doi: 10.1186/s12916-020-01854-w (PMC7724883; doi:10.1186/s12916-020-01854-w)
Supplement: Supplementary file 1 — Additional file 1: Table S1. Drug and diagnosis codes used in the study. [file 12916_2020_1854_MOESM1_ESM.docx]

**Table S1.** Drug and diagnosis codes used in the study

| **Drug** | **ATC codes** |
| --- | --- |
| Antidepressants^†^ | N06AA, N06AB, N06AX, N06AF, N06AX12, N06AX11, N06AX14, N06AX05 |
| Benzodiazepines^‡^ | N03AE, N05BA, N05CD |
| Angiotensin converting enzyme inhibitors | C09A, C09B |
| Angiotensin II receptor blockers | C09C, C09D |
| Anticholinergics | N04A |
| Anticoagulants | B02BD |
| Antiplatelet inhibitors | B01AC |
| Antipsychotics | N05A |
| Anticonvulsants | N03A |
| Digoxin | C01AA05 |
| Non-insulin glucose lowering agents | A10B |
| Anti-inflammatory analgesics | N02B |
| β-blockers | C07 |
| Calcium channel blockers | C08 |
| Insulin | A10A |
| Lipid lowering agents | C10 |
| Narcotic analgesics | N02 |
| Nonsteroidal anti-inflammatory drugs | M01A |
| Other anxiolytics^‡⁋^ | N05B |
| Thiazide diuretics | C03A |
| **Diagnosis** | **ICD-10 codes** |
| Depression | F32, F33, F34.1 |
| Suicide attempt/self-harm | X60-X84, Y870 |
| Manic episode | F30 |
| Bipolar disorder | F31 |
| Anxiety | F40, F41 |
| Insomnia | G47, F51 |
| Substance abuse | F10-F19 |
| Cancer | C00-C99 |
| Cerebrovascular disease | I60-I69, G45 |
| Chronic kidney disease | N03.2-N03.7, N05.2-N05.7, N18-N19, N25.0, Z49.0-2, Z94.0, Z99.2 |
| Chronic obstructive pulmonary disease | J41-J44 |
| Dementia | F00-F03, F05.1, G30-G31 |
| Diabetes mellitus | E10-E14 |
| Epilepsy | G40-G41 |
| Fracture | S22.0, S22.1, S27.0, S27.1, S32.0, S42.2, S42.3, S52.5, S52.6, M48.4, M48.5, T79, T98, V01-V99, W11-W17, W50-W52, W64, X34-X39 |
| Hypertension | I10-I15 |
| Hyperlipidemia | E78 |
| Ischemic heart disease | I24-I25 |
| Osteoarthritis | M15-M19 |
| Parkinson’s disease | G20 |
| Rheumatoid arthritis | M05-M06 |

**Note:** ATC, Anatomical Therapeutic Chemical classification code; ICD-10, International Classification of Disease, 10th Revision codes

^†^Antidepressants included were moclobemide, toloxatone, mirtazapine, bupropion, nefazodone, trazodone, venlafaxine, milnacipran, duloxetine, desvenlafaxine, tianeptine, fluoxetine, fluvoxamine, paroxetine, sertraline, sertraline, citalopram, escitalopram, amineptine, amitriptyline, amoxapine, clomipramine, dosulepin, doxepin, imipramine, maprotiline, nortriptyline, quinupramine

^‡^Benzodiazepines included were alprazolam, bromazepam, brotizolam, chlordiazepoxide, clobazam, clorazepate, clotiazepam, diazepam, estazolam, ethyl loflazepate, etizolam, fludiazepam, flurazepam, flunitrazepam, lorazepam, midazolam, nordazepam, oxazepam, pinazepam, tofisopam, triazolam

^⁋^Other anxiolytics included were tandospirone citrate, hyperici herba, medifoxamine, mianserin, minaprine, agomelatine, vortioxetine, hydroxyzine HCl, buspirone HCl, etifoxine
