# Supplementary figures and images for: Risk of mortality associated with concomitant antidepressant and benzodiazepine therapy among patients with depression: a population-based cohort study
Source: BMC Med. 2020 Dec 9;18:387. doi: 10.1186/s12916-020-01854-w (PMC7724883; doi:10.1186/s12916-020-01854-w)

**Fig. S1.** Overall study design


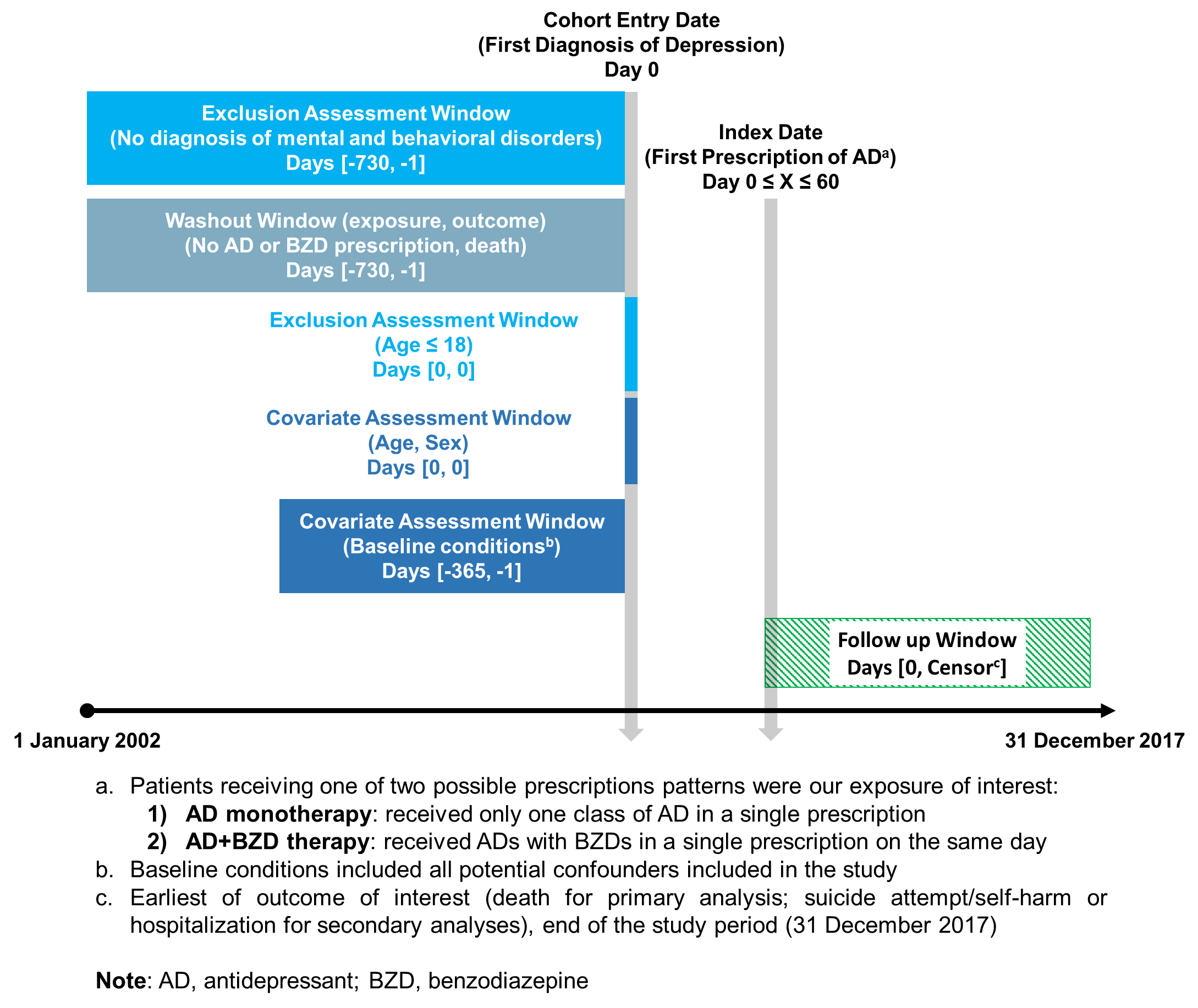

Supplement: Supplementary file 2 — Additional file 2: Fig. S1. Overall study design. [file 12916_2020_1854_MOESM2_ESM.docx]

**Fig. S2.** Exposure classification

**
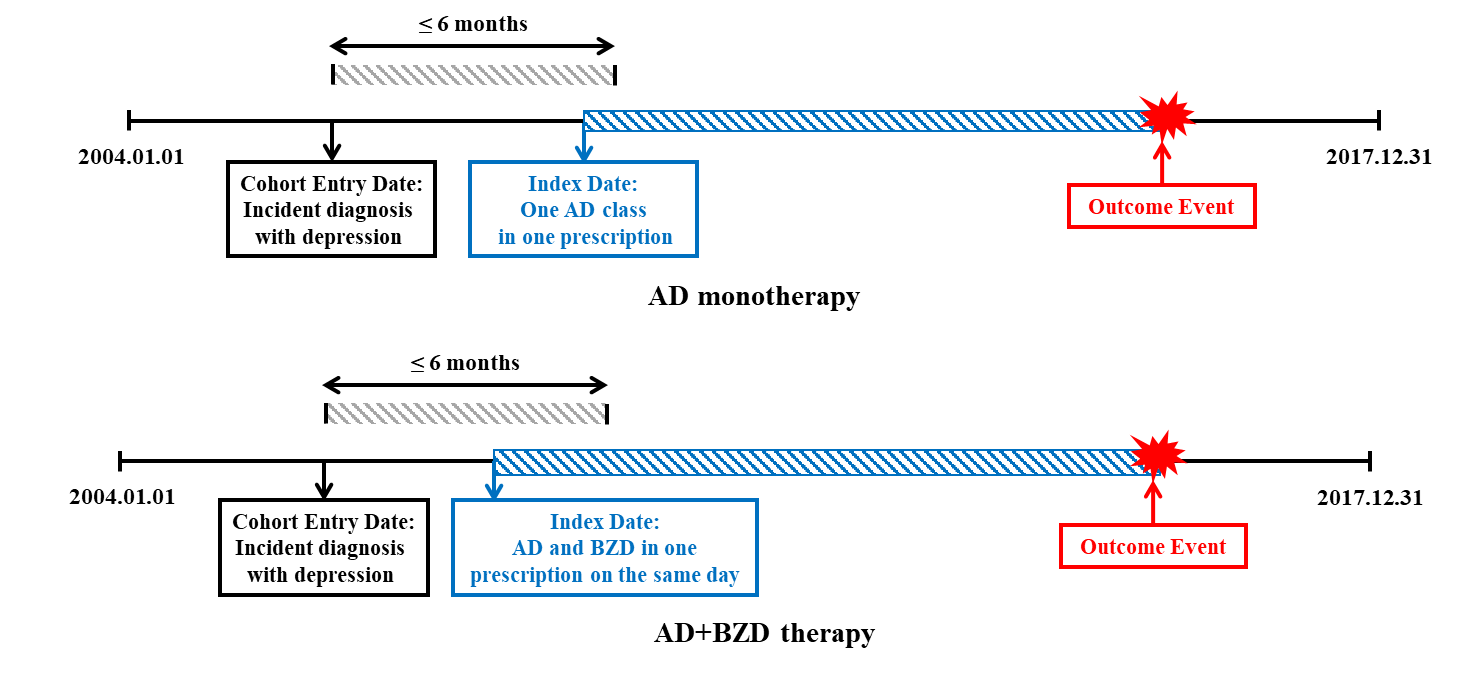
**

Supplement: Supplementary file 3 — Additional file 3: Fig. S2. Exposure classification. [file 12916_2020_1854_MOESM3_ESM.docx]

**Fig. S3.** Varying the definition of the time-window for concomitant therapy

**
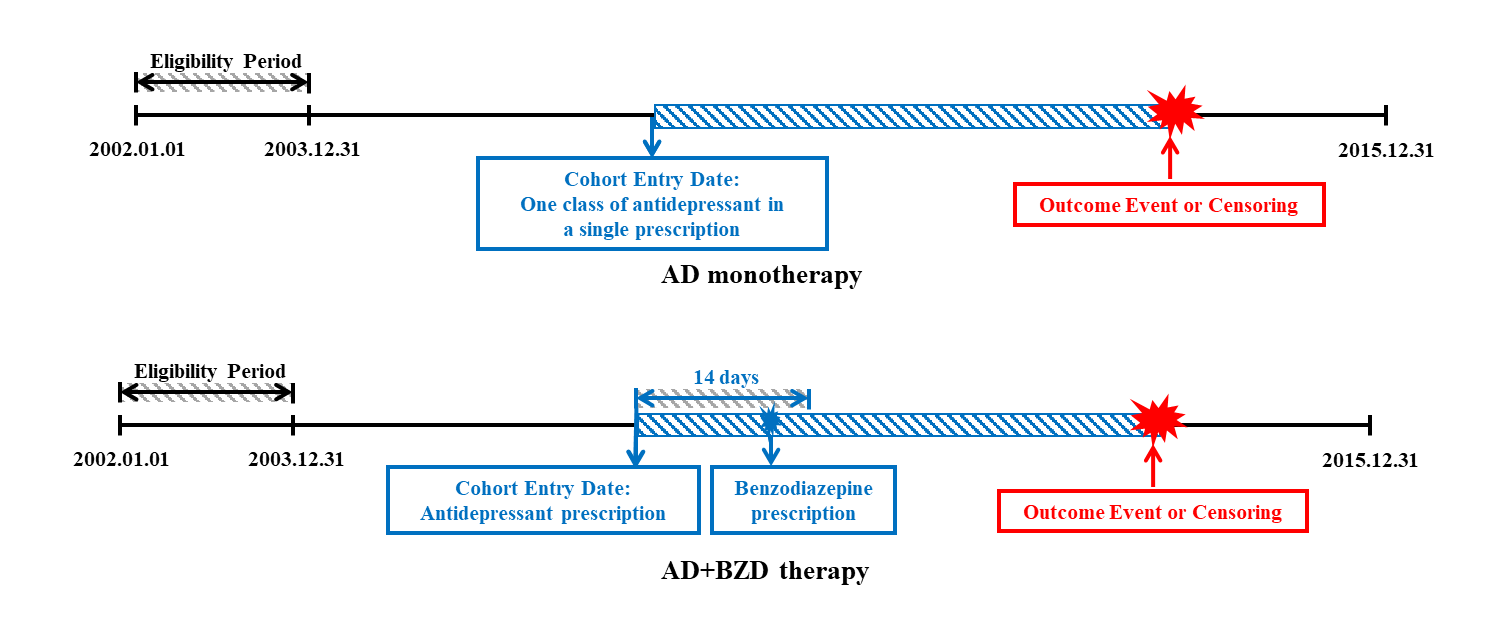
**

Supplement: Supplementary file 4 — Additional file 4: Fig. S3. Varying the definition of the time-window for concomitant therapy. [file 12916_2020_1854_MOESM4_ESM.docx]
