# Supplementary material for: Risk of mortality associated with concomitant antidepressant and benzodiazepine therapy among patients with depression: a population-based cohort study
Source: BMC Med. 2020 Dec 9;18:387. doi: 10.1186/s12916-020-01854-w (PMC7724883; doi:10.1186/s12916-020-01854-w)
Supplement: Supplementary file 5 — Additional file 5: Table S2. Baseline characteristics of study subjects before propensity score matching, where values are percentages unless stated otherwise. [file 12916_2020_1854_MOESM5_ESM.docx]

**Table S2.** Baseline characteristics of study subjects before propensity score matching, where values are percentages unless stated otherwise

|  | | **Cohort before propensity score matching** | | | | |
| --- | --- | --- | --- | --- | --- | --- |
|  |  | **N = 612,729 (%)** | | | | |
|  |  | **AD monotherapy** | | **AD+BZD therapy** | | **aSD** |
|  |  | **N = 276,045 (%)** | | **N = 336,684 (%)** | |  |
| **Follow-up (years; mean ± std)** | | 5.47 | ±3.84 | 6.30 | ±4.15 | 0.206 |
| **Age (years; mean ± std)** | | 45.4 | ±18.0 | 41.5 | ±15.5 | 0.234 |
| **Male** | | 121,185 | 43.90 | 133,808 | 39.74 | 0.084 |
| **Type of insurance** | |  |  |  |  | 0.000 |
|  | Healthcare insurance | 265,175 | 96.06 | 324,291 | 96.32 |  |
|  | Medical aid | 10,642 | 3.86 | 12,106 | 3.60 |  |
| **Residential district** | |  |  |  |  | 0.055 |
|  | Metropolitan | 149,444 | 54.14 | 174,297 | 51.77 |  |
|  | Urban | 45,909 | 16.63 | 63,655 | 18.91 |  |
|  | Rural | 80,010 | 28.98 | 98,048 | 29.12 |  |
| **Income level** | |  |  |  |  | 0.074 |
|  | 1^st^ quartile | 48,555 | 17.59 | 64,877 | 19.27 |  |
|  | 2^nd^ quartile | 53,208 | 19.28 | 71,253 | 21.16 |  |
|  | 3^rd^ quartile | 66,183 | 23.98 | 81,153 | 24.10 |  |
|  | 4^th^ quartile | 91,191 | 33.03 | 99,956 | 29.69 |  |
| **Charlson Comorbidity Index (mean ± std)** | | 0.41 | ±0.85 | 0.33 | ±0.75 | 0.198 |
|  | 0 | 208,412 | 75.50 | 268,831 | 79.85 |  |
|  | 1 | 34,310 | 12.43 | 36,846 | 10.94 |  |
|  | 2 | 22,353 | 8.10 | 22,024 | 6.54 |  |
|  | 3 | 10,326 | 3.74 | 8,335 | 2.48 |  |
|  | 4 | 154 | 0.06 | 204 | 0.06 |  |
|  | ≥5 | 490 | 0.18 | 444 | 0.13 |  |
| **Comorbidities^†^** | |  |  |  |  |  |
|  | Anxiety | 4,903 | 1.78 | 7,306 | 2.17 | 0.028 |
|  | Cancer | 4,491 | 1.63 | 3,856 | 1.15 | 0.041 |
|  | Cerebrovascular disease | 7,790 | 2.82 | 4,676 | 1.39 | 0.100 |
|  | Chronic kidney disease | 933 | 0.34 | 646 | 0.19 | 0.028 |
|  | Chronic obstructive pulmonary disease | 10,438 | 3.78 | 10,336 | 3.07 | 0.039 |
|  | Dementia | 6,724 | 2.44 | 2,013 | 0.60 | 0.151 |
|  | Diabetes mellitus | 19,628 | 7.11 | 16,478 | 4.89 | 0.093 |
|  | Epilepsy | 1,876 | 0.68 | 1,080 | 0.32 | 0.051 |
|  | Fractures | 1,709 | 0.62 | 1,523 | 0.45 | 0.023 |
|  | Hypertension | 35,337 | 12.80 | 30,513 | 9.06 | 0.120 |
|  | Hyperlipidemia | 32,964 | 11.94 | 28,931 | 8.59 | 0.110 |
|  | Insomnia | 8,164 | 2.96 | 12,917 | 3.84 | 0.049 |
|  | Ischemic heart disease | 2,170 | 0.79 | 1,629 | 0.48 | 0.038 |
|  | Osteoarthritis | 27,526 | 9.97 | 22,968 | 6.82 | 0.114 |
|  | Parkinson’s disease | 537 | 0.19 | 203 | 0.06 | 0.038 |
|  | Rheumatoid arthritis | 4,709 | 1.71 | 3,793 | 1.13 | 0.049 |
|  | Substance abuse | 965 | 0.35 | 2,238 | 0.66 | 0.044 |
| **History of medication use^†^** | |  |  |  |  |  |
|  | Angiotensin converting enzyme inhibitors | 2,591 | 0.94 | 2,218 | 0.66 | 0.031 |
|  | Angiotensin II receptor blockers | 19,756 | 7.16 | 16,152 | 4.80 | 0.100 |
|  | Anticholinergics | 28,679 | 10.39 | 32,399 | 9.62 | 0.026 |
|  | Antiplatelets & anticoagulants | 26,050 | 9.44 | 20,727 | 6.16 | 0.123 |
|  | Antipsychotics | 2,319 | 0.84 | 2,983 | 0.89 | 0.001 |
|  | Anticonvulsants | 7,386 | 2.68 | 4,523 | 1.34 | 0.095 |
|  | Digoxin | 553 | 0.20 | 353 | 0.10 | 0.024 |
|  | Non-insulin glucose lowering agents | 11,728 | 4.25 | 9,430 | 2.80 | 0.079 |
|  | Anti-inflammatory analgesics | 109,792 | 39.77 | 12,6442 | 37.56 | 0.046 |
|  | β-blockers | 12,084 | 4.38 | 12,536 | 3.72 | 0.033 |
|  | Calcium channel blockers | 21,095 | 7.64 | 18,306 | 5.44 | 0.089 |
|  | Insulin | 1,291 | 0.47 | 937 | 0.28 | 0.031 |
|  | Lipid lowering agents | 18,842 | 6.83 | 12,656 | 3.76 | 0.094 |
|  | Narcotic analgesics | 69,754 | 25.27 | 74,673 | 22.18 | 0.073 |
|  | Nonsteroidal anti-inflammatory drugs | 151,262 | 54.80 | 175,902 | 52.25 | 0.051 |
|  | Other anxiolytics | 16,454 | 5.96 | 19,054 | 5.66 | 0.013 |
|  | Thiazide diuretics | 12,926 | 4.68 | 11,385 | 3.38 | 0.066 |

**Note**: AD, antidepressant; aSD, absolute standardized difference; BZD, benzodiazepine; std, standard deviation.

**^†^**Assessed within the year before cohort entry
