# Supplementary material for: Risk of mortality associated with concomitant antidepressant and benzodiazepine therapy among patients with depression: a population-based cohort study
Source: BMC Med. 2020 Dec 9;18:387. doi: 10.1186/s12916-020-01854-w (PMC7724883; doi:10.1186/s12916-020-01854-w)
Supplement: Supplementary file 6 — Additional file 6: Table S3. Top 20 frequent causes of hospitalization by ICD-10 diagnosis code to 3 places. [file 12916_2020_1854_MOESM6_ESM.docx]

**Table S3.** Top 20 frequent causes of hospitalization by ICD-10 diagnosis code to 3 places

| **ICD-10** | **Condition** | **Frequency** |
| --- | --- | --- |
| F32 | Depressive episode | 8,494 |
| M51 | Other intervertebral disc disorders | 8,399 |
| I84 | Hemorrhoids | 7,831 |
| O80 | Single spontaneous delivery | 6,050 |
| F10 | Mental and behavioral disorders due to use of alcohol | 4,847 |
| A09 | Other gastroenteritis and colitis of infectious and unspecified origin | 4,839 |
| O82 | Single delivery by caesarean section | 3,973 |
| K35 | Acute appendicitis | 3,283 |
| D25 | Leiomyoma of uterus | 3,115 |
| I63 | Cerebral infarction | 2,579 |
| M50 | Cervical disc disorders | 2,516 |
| M75 | Shoulder lesions | 2,479 |
| M48 | Other spondylopathies | 2,461 |
| J18 | Pneumonia, organism unspecified | 2,435 |
| S33 | Dislocation, sprain and strain of joints and ligaments of lumbar spine and pelvis | 2,348 |
| F20 | Schizophrenia | 2,174 |
| S83 | Dislocation, sprain and strain of joints and ligaments of knee | 2,162 |
| M17 | Gonarthrosis [arthrosis of knee] | 2,147 |
| I20 | Angina pectoris | 2,120 |
| S82 | Fracture of lower leg, including ankle | 2,093 |

Note: ICD-10, International Classification of Diseases, 10^th^ Revision
