# Supplementary material for: Risk of mortality associated with concomitant antidepressant and benzodiazepine therapy among patients with depression: a population-based cohort study
Source: BMC Med. 2020 Dec 9;18:387. doi: 10.1186/s12916-020-01854-w (PMC7724883; doi:10.1186/s12916-020-01854-w)
Supplement: Supplementary file 7 — Additional file 7: Fig. S4. Forest plot summarizing the results of sensitivity analyses for the risk of all-cause mortality. [file 12916_2020_1854_MOESM7_ESM.docx]

**Fig. S4.** Forest plot summarizing the results of sensitivity analyses for the risk of all-cause mortality


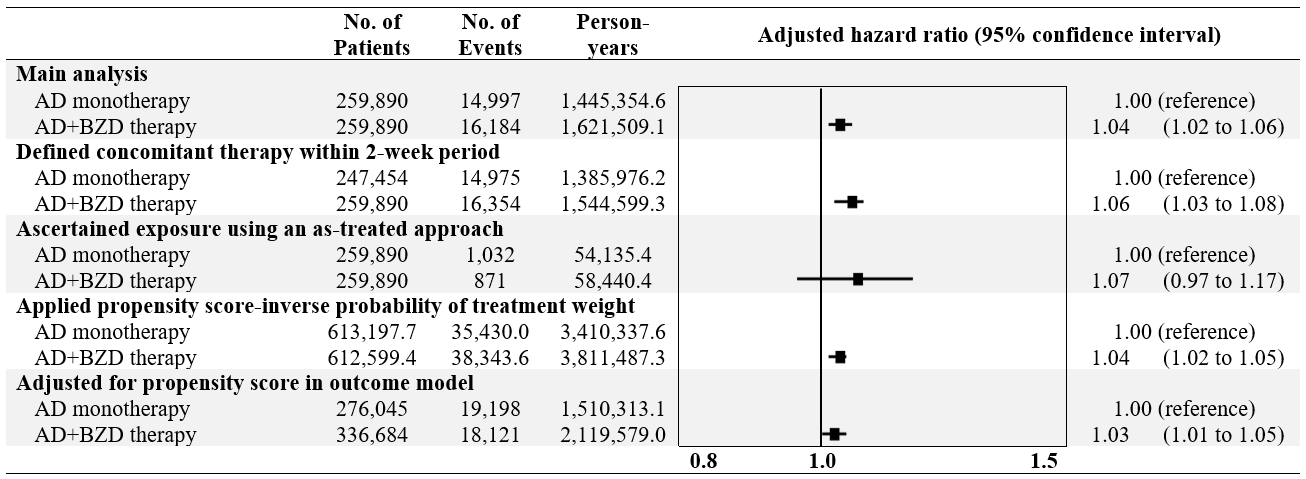


**Note:** AD, antidepressants; BZD, benzodiazepines
