# Supplementary material for: Risk of mortality associated with concomitant antidepressant and benzodiazepine therapy among patients with depression: a population-based cohort study
Source: BMC Med. 2020 Dec 9;18:387. doi: 10.1186/s12916-020-01854-w (PMC7724883; doi:10.1186/s12916-020-01854-w)
Supplement: Supplementary file 8 — Additional file 8: Table S4. Results of sensitivity analyses that examined the effects from unmeasured confounders on the association between risk of mortality and the use of antidepressants with benzodiazepines versus antidepressants alone. [file 12916_2020_1854_MOESM8_ESM.docx]

**Table S4.** Results of sensitivity analyses that examined the effects from unmeasured confounders on the association between risk of mortality and the use of antidepressants with benzodiazepines versus antidepressants alone

|  | **HR** | **95% CI** | **E-value**^†,⁋^ **for HR** | **E-value**^†,⁋^ **for CI** |
| --- | --- | --- | --- | --- |
| AD+BZD therapy | 1.04 | 1.02 (LL) | 1.24 | 1.16 |

**Note:** CI, confidence interval; HR, hazard ratio; LL, lower limit; UL, upper limit

^†^Conditional on measured covariates, the E-value is the minimum strength of association that an unmeasured confounder needs to have with the exposure and outcome to explain the observed association.

^⁋^Calculating the E-values for HR or CI,

| HR > 1 |  |  |
| --- | --- | --- |
|  | HR | E-value = HR + √[HR x (HR - 1)] |
|  | CI | If LL of CI ≤ 1, then E-value = 1 |
|  |  | If LL of CI > 1, then E-value = LL + √[LL x (LL - 1)] |
| HR < 1 |  |  |
|  | HR | E-value = (1 / HR) + √[(1 / HR) x (1 / HR) - 1)] |
|  | CI | If UL of CI ≥ 1, then E-value = 1 |
|  |  | If UL of CI < 1, then E-value = (1 / UL) + √[(1 / UL) x ((1 / UL) - 1)] |
